# Recent Advances in Amyloids Structural Studies and Thin Film Applications

**DOI:** 10.3390/molecules30142908

**Published:** 2025-07-09

**Authors:** Eugenia Pechkova, Stefano Fiordoro, Alberto Izzotti, Christian Riekel

**Affiliations:** 1 Laboratories of Biophysics and Nanotechnology, Department of Experimental Medicine (DIMES), University of Genova Medical School, Via A. Pastore, 3, 16132 Genova, Italy; 2 Department of Health Science (DISSAL), University of Genova Medical School, 16132 Genova, Italy; stefano.fiordoro@edu.unige.it; 3 Department of Experimental Medicine (DIMES), University of Genova Medical School, 16132 Genova, Italy; alberto.izzotti@unige.it; 4 IRCCS Ospedale Policlinico San Martino, Largo Rosanna Benzi 10, 16132 Genova, Italy; 5 European Synchrotron Radiation Facility, CS40220, F-38043 Grenoble Cedex 9, France; riekel@esrf.fr

**Keywords:** amyloid fibrils, amyloid thin film, high brilliance X-ray diffraction, cryo-electron microscopy

## Abstract

Amyloids are protein-based biomaterials composed of fibrils with cross-β cores. Previously only associated with degenerative diseases, such as Parkinson’s disease, Alzheimer’s disease, and diabetes, amyloids remain active and functional both in vivo and in vitro conditions, enabling a variety of applications in medicine, nanotechnology, and biotechnology. This review aims to review the most advanced methods for amyloid fibril structural studies, with special attention on amyloid thin films. Selected advances of biomedical and biotechnological relevance will be outlined, and perspectives for future studies in the context of ongoing methodological progress will be discussed.

## 1. Introduction

Neurodegenerative diseases affect more than 40 million people worldwide. At the molecular level, these diseases are caused by the aggregation of proteins into soft biomaterials composed of insoluble fibrils called amyloids. Amyloids are usually associated with Alzheimer’s, Parkinson’s, and Huntington’s diseases; type II diabetes; prion-transmissible disorders; and others [1]. Research on protein aggregation, and specifically amyloid buildup in organs called amyloidosis, has in recent years attracted a lot of attention. Indeed, a rising number of functional proteins have been found that are capable of forming an amyloid state, biologically benefitting their host [2]. It is now known that amyloids can play several functions [3,4] in different biological processes, ranging from hormone storage to necroptosis of cells [5]. The amyloid state is accessible under certain conditions to multiple proteins and can therefore be considered as an intrinsic property of amino acid sequences [6]. Indeed, globular proteins reach the amyloid state upon denaturation while forming fibrils composed of elongated stacks of β-strands, stabilized by hydrogen bonds. The so-called “cross-β structure” has the strands aligned perpendicular to the fibril axis with an inter-strand distance of 4.7 Å, which rises from the X-ray diffraction meridional reflection and corresponds to the hydrogen-bonding between paired carbonyl and amide groups in adjacent β-strands. Moreover, X-ray diffraction from amyloid fibrils shows a characteristic equatorial reflection at 6–11 Å, corresponding to the distance between stacked β-sheets [6,7]. The fibrils undergo a higher-order self-assembly into twisted ropes or tapes and are usually composed of one or more protofilaments with diameters of several nanometers [8]. Aggregation of these supramolecular structures in living organisms can result in plaques causing a number of neurodegenerative diseases, while similar in vitro aggregation of non-disease-associated proteins appears to be highly cytotoxic [9]. It is worthwhile noting that intermediate aggregates appear to be more responsible for in vivo toxicity than the fibrillar products themselves [10], although the inert state of the fibrillar deposits has been challenged.

Amyloid fibers are characterized by remarkable stability, mechanical rigidity, and resistance to breakage, resembling in many ways synthetic polymers. Their self-assembly properties are also surprising, allowing, for example, complete reassembly upon denaturation [11,12]. In various applications, they can act as structural scaffolds [13,14], catalysts [15,16,17], and functional coatings [18,19].

In this review, we will outline recent progress in amyloid structural studies and pioneering discoveries concerning amyloid fibrils pertinent both for medical and for bio- and nanoscience, with special attention to the amyloid assembly in nanofilms, opening new avenues for their future studies and applications. Our main goal is not to provide an exhaustive overview of studies on amyloid fibrillation mechanisms and specific structural features but to report on forefront works and discuss their impact and future prospects.

## 2. Amyloid’s Advanced Characterization Methods

The heterogeneity of amyloid fibrils and their nanoscale organization pose challenges to studying amyloid formation processes and developing models for the molecular mechanisms of amyloid-related pathologies. It was soon recognized that the transition of globular proteins into elongated intermediates during amyloid formation depends on a number of factors such as pH [20], concentration [21], temperature [22], and post-translational modifications (PTMs) [23]. The initial assumption of a nucleation–polymerization process playing a role during amyloid formation has now been replaced by a number of concurrent processes [24,25,26]. Despite the fact that all amyloid fibrils have a similar β-sheet structure, there are many different ways for them to develop. Indeed, denatured proteins that are fully or partially folded, preexisting fibers (seeding), and/or amorphous aggregates can all start the process of oligomerization and fibrillogenesis. The kinetics and mechanics of aggregation are very closely related. Triggering factors such as temperature, concentration, pH, and a variety of interaction mechanisms (such as seeding, co-aggregation, metal ion presence, etc.) are all important determinants [24].

The complexity of amyloid formation requires careful visualization and characterization of the fibrillar state. Fluorescence and atomic force microscopy (AFM) techniques are routinely used in laboratories. Indeed, recent developments in amyloid imaging by a fluorescent amyloid sensor allow distinguishing fibrils with different morphologies with a resolution of ≈30 nm, enabling visualization and quantification of morphological characteristics such as the length and skew of individual amyloid aggregates that are generated at various points along the amyloid assembly route [27]. Single-molecule fluorescence microscopy allows ultrasensitive detection and visualization of individual amyloid fibrils and oligomers by a method known as single-aggregate visualization by enhancement (SAVE) [28,29]. High-resolution imaging can also be performed by AFM [30], providing nanoscale lateral resolution and sub-nanometer vertical resolution of amyloid plaques. Additionally, post-imaging analysis can be used to retrieve parameters such as nanoscale roughness of amyloid networks [31]. AFM techniques allow probing in vitro the evolution of hierarchical organization, manipulating individual fibrils or protofilaments, as well as their mechanical characterization [32,33].

**Figure 1:**
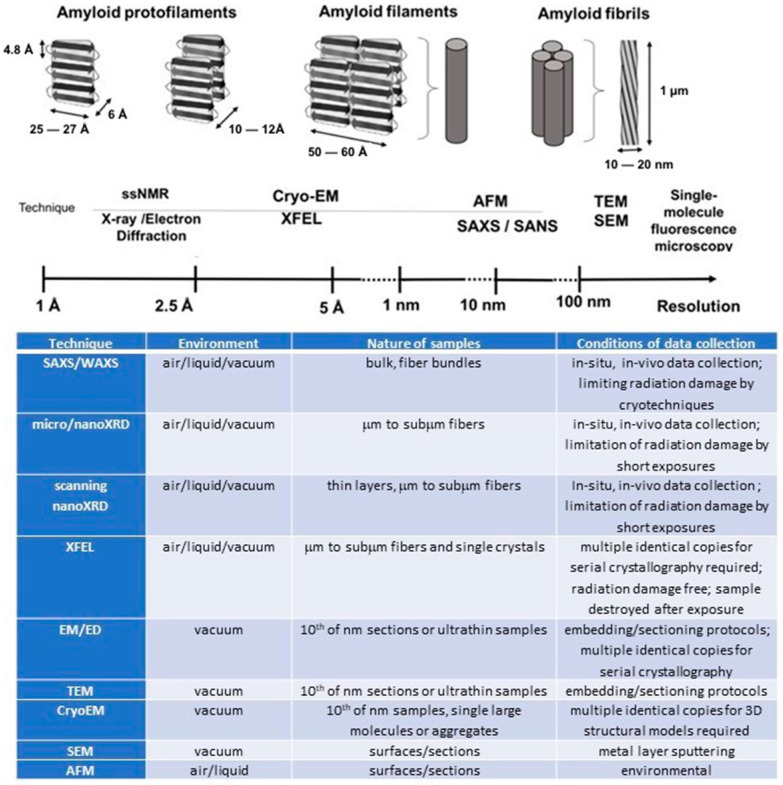
Resolution scale, sample environment, and data collection conditions for different techniques for imaging and structural studies of amyloids.

It is noteworthy that the ability to characterize the structure of complex biological molecules and macromolecular complexes has substantially improved as a result of recent technological advancements. Numerous other advanced techniques now shed new light on the amyloid structure at the atomic level, revealing a variety of polymorphic structures that generally fit the cross-β amyloid motif (Figure 1). Indeed, the rational design of new therapeutic medicines is aided by the understanding of the three-dimensional structure of amyloid fibrils from various origins, which offers significant insights into the mechanics of amyloid development [34]. The combination of structural biology data from various methodologies and experimental techniques offers fresh perspectives on the atomic-level resolution formation of fibrillar structures by individual protein subunits [35]. In Figure 1, the sample environment, nature of samples, and conditions of data collection are indicated for every experimental technique.

Solid-state nuclear magnetic resonance (ssNMR) has proven to be a highly effective technique to unravel the overall structure of fibrils. The ssNMR technique requires expressing recombinant amyloid-forming proteins in a medium containing isotopically labeled amino acids, followed by the formation of fibrils and the detection of resonances [36,37,38,39]. Innovations in high-field magnets, pulse sequences, high-resolution multi-channel magic-angle spinning ultrafast probes result in reliable models illustrating the overall conformation of the protofilament spine chains. The quality of the models, such as sensitivity and spectral resolution, deteriorates with increasing molecule weight.

X-ray diffraction (XRD) and electron diffraction (ED) techniques can be used for studying the atomic structure of amyloid crystals and fibers [40,41,42], while small-angle X-ray scattering (SAXS) targets specifically nano- and meso-scale assemblies [43,44,45]. Single-crystal XRD techniques making use of high-brilliance X-ray beams from synchrotron radiation (SR) or X-ray free-electron laser (XFEL) sources in combination with single-photon-sensitive pixel detectors provide high-resolution, three-dimensional (3D) amyloid structures based on multiple crystals (so-called “serial crystallography”) [46]. This reveals, however, only the crystalline β-sheet-forming core, while disordered features remain hidden. Ultrabrilliant, femtosecond (fs) XFEL pulses allow avoiding radiation damage issues inherent in high-resolution SR diffraction and provide access to diffraction patterns from single amyloid fibrils [47]. XFEL experiments also provide access to ultrafast structural kinetics [47,48] but are technically challenging and require considerable resources. The strong development of remote beamline attendance and data analysis during the coronavirus pandemic has allowed, however, reducing external staff required for performing experiments at an XFEL facility [49]. The advent of a new generation of compact XFEL sources should also provide more beamtime dedicated to biological studies [49]. We note that SR and XFEL experiments can be performed in microfluidic environments, in principle close to physiological conditions. Indeed, amyloid fibrillation has been observed at the rim of drying droplets of peptide solutions by SR techniques [50].

Recent developments in cryo-electron microscopy (Cryo-EM) allow obtaining near-atomic-resolution structures of macromolecular complexes without the requirement for crystals. An advanced Cryo-EM detector allows recording incident electrons in a thin, sensitive layer so that the signal is not scattered into neighboring pixels, improving image processing and reaching the XRD crystallography resolution [51]. Four-dimensional structures can be generated by reconstructing views of various molecular orientations. Cryo-EM can produce the overall fibril structure, allowing us to obtain information on protofilaments, the amount of twist, and, depending on the quantity of well-ordered specimens, data on the atomic structure of the fibril by merging information from many identical fibrils [52,53,54,55,56]. Cryo-EM methodology is advancing quickly, becoming a widely used approach for 3D structures with atomic resolution.

In general, Cryo-EM techniques are more accessible to the scientific community than SR or XFEL techniques, although sample preparation is still rather difficult in terms of sample thickness or particle distribution, and the most advanced Cryo-EM instruments are rather expensive. Several SR facilities provide Cryo-EM set-ups for external users. As to the most recent results, fascinating research has revealed the role of neuronal functional amyloids in memory persistence, based on functional Cryo-EM structure amyloid aggregates that stabilize long-term memory [56]. Combining information from several complementary studies, therefore, allows obtaining more accurate models of the amyloid structures. Indeed, precise crystallographic crystal structures of fragments can be fitted into a Cryo-EM reconstruction, producing highly informative pseudoatomic models [56]. While NMR allows a local reconstruction of small repeating units of the fibril, leaving out long-range fiber packing and twists, Cryo-EM reconstructions make it possible to image whole fibers, but not individual protofilaments. XFEL-based experiments have the potential to record diffraction from individual protofilaments and build upon existing results from solid-state NMR and Cryo-EM, contributing to enhanced comprehension of the individual protofilaments’ structures. According to Lashuel et al. [57], protofilaments, composed of bilayered, 25–27 Å wide and 10–12 Å high cross β-structures, are at the base of amyloid hierarchical structural organization. Indeed, protofilaments self-assemble into 50–60 Å wide filaments and are assumed to form fibrils characterized by 100–130 Å (2- or 4-filament) structures, which then self-assemble into fibers or ribbon-like assemblies, i.e., macroscopic aggregates of multiple fibrils [58].

## 3. Detection of Amyloids in Selected Recent Biomedical Research

The principal aim of biomedical research is the detection and destruction of amyloid fibrils and plaques in the human body. This requires a deep understanding of the mechanism of amyloid fibrillation, their exact composition, and neurotoxicity for the diagnosis and treatment of these conditions. However, it is still unclear how amyloids aggregate and cause the death of neurons. Indeed, the low concentration of soluble amyloid precursor proteins in human biofluids and their high degree of variability have not allowed precisely determining their role during neurodegenerative disorders [59]. Detection of amyloid aggregates in vivo and in vitro lies at the basis of diagnostic and therapeutic approaches. Several techniques have been developed to detect amyloid aggregates, including electrochemical approaches [60], fluorescence methods [61], colorimetric methods [62], surface plasmon resonance (SPR) and surface enhanced Raman spectroscopy (SERS) [63,64], enzyme-labeled immunosorbent assay (ELISA) [65], quartz crystal microbalance (QCM), and other types of sensors [66].

The destabilization of hydrogen bonds in β-rich structures is generally the main focus of the design of prospective therapeutic agents and medications. Since ferritin derivatives could destabilize fibril structures, they have been suggested for various upcoming medical applications [67]. Reconstructed ferritin (RF) and magnetoferritin (MF) have, however, been shown to have a damaging effect on lysosome amyloid fibrils, confirmed by AFM and fluorescence spectrophotometry [68]. It was determined that iron was the primary cause of protein amyloid fibril destruction. On the other hand, the presence of RF and MF causes a significant increase in potentially toxic ferrous ions in the body.

Another approach is to capture and precipitate amyloid-containing seed aggregates from human cerebrospinal fluid (CSF) in order to better understand how the various types of soluble aggregates present in biofluids of patients with Parkinson’s disease and Alzheimer’s disease may affect the development of pathologies [69]. In order to identify and target oligomeric amyloid precursor molecules that are present in more-or-less complex body fluids such as human CSF, a new class of Y-shaped small molecules called CAP-1 has been developed to precipitate amyloids. CAP-1 has two binding sites, one of which is specific for the amyloid β-sheets prevalent in Parkinson’s and Alzheimer’s diseases, and the other is connected to biotin to immobilize the resulting structure on a magnetic bead surface. Thioflavin T, a fluorescent probe, was used to recognize the aggregate binding. In order to collect amyloid aggregates in CSF, CAP-1 was first attached to magnetic beads. Total internal reflection and atomic force microscopy were used to establish the presence of amyloids. Next, high-resolution mass spectrometry was used to identify protein aggregates, and it was shown that CAP-1-attached proteins had higher β-strand content than ordinary beads did. It has therefore become possible to target the amyloid structure of all protein aggregates found in human fluids, to isolate them for examination, and to characterize them using single-molecule fluorescence imaging and mass spectrometry. The molecular composition and structural characteristics of in vivo aggregates generated in neurodegenerative diseases can thus be determined using amyloid precipitation. Further studies could pave the way to early diagnosis and therapy of neurodegenerative diseases.

A further promising method is based on magnetic nanoparticles (MNPs), well known due to their great potential in biomedical and clinical applications. Primarily employed in the treatment of amyloid disorders, MNPs were recently used for the detection and quantification of amyloid aggregates. With their capacity to regulate the aggregation of amyloidogenic proteins, MNPs can contribute to understanding amyloid fibrillation processes. The functionalization of several MNPs’ surfaces by various moieties (molecules, peptides, antibody fragments, or complete MNP antibodies) can serve for the detection and quantification of amyloid aggregates by magnetic-resonance-based amyloid imaging in biomedical fields [70].

In conclusion, even if amyloids are historically associated with cell disorder, much evidence confirms that functional amyloids are the rule rather than the exception in cellular biology [71]. Organisms purposefully produce functional amyloids in order to take advantage of their numerous functional characteristics. As a result, a lot of attention was dedicated to biomedical applications of the amyloids. Antiviral activity of amyloid peptides against human viral infections and cell-penetrating amyloid peptides that act as amyloidogenesis inhibitors and drug delivery carriers are just a few examples [72].

## 4. Amyloids as Functional Materials for Selected Technological Applications

In recent decades, a lot of studies have been dedicated to those key characteristics of amyloid fibrils that make them functional materials in nature. Indeed, the emergence of function from protein self-assembly at various length scales is best shown by amyloid materials. The relationships between mesoscale structure and material function were particularly emphasized, and it was shown how naturally occurring examples of functional amyloids can shed light on potential future uses of synthetic amyloid-based materials [73].

Potential applications of amyloid supramolecular assemblies exceed those of synthetic polymers since the building blocks can introduce biological function in addition to mechanical properties or catalytic activity [74,75]. Combining mechanical and catalytic properties of amyloid fibrils allows developing new avenues for nanobiomaterials [76,77,78]. Indeed, amyloid fibrils could be designed for next-generation sustainable applications with low impact on the environment, such as the following: (i) sustainable and efficient water purification [79]; (ii) adsorbents for an effective removal of heavy metals from wastewater [80]; and (iii) designing materials with tailored applications based on the known amyloid atomic structure, allowing, for example, capturing carbon dioxide from flue gas [81], which addresses the global problem of excess anthropogenic carbon dioxide.

### 4.1. Amyloid Thin Film Production and Characterization

**Figure 2:**
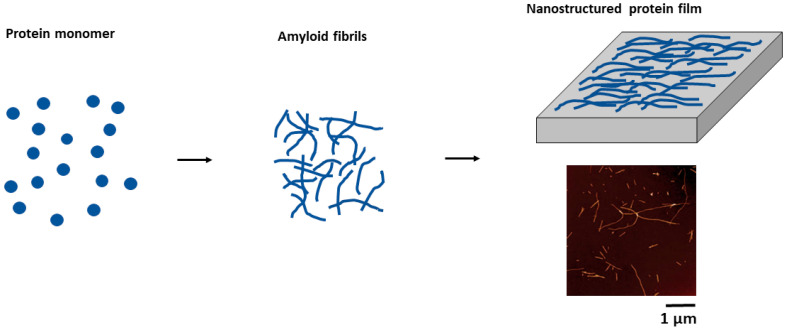
Protein molecules are first assembled into amyloid fibrils, which are then stacked into thin films. Atomic force micrograph of the lysozyme fibrils is shown here (adapted from Knowles et al. [84]).

**Figure 3:**
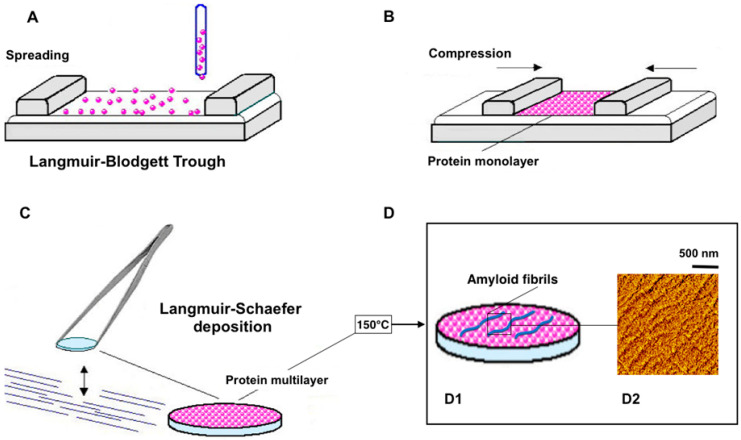
Amyloid fibrils obtained in the protein Langmuir–Blodgett multilayers, prepared by the Langmuir–Blodgett technique (**A**–**C**), followed by 150 °C thermal annealing (**D**), resulting in fibrillation (**D1**) evident on atomic force micrograph of the phycocyanin-annealed thin film (**D2**).

With an increasing interest in amyloids and their application in science and technology, the development of methods allowing their immobilization for specific applications has become important. Methods for fabricating amyloid thin films and their characterization have become an important R&D area, opening new avenues for multiple applications [82]. Indeed, immobilization of functional amyloid into hybrid membranes provides new opportunities for continuous flow catalysis [83]. In the following, we will provide an overview of the two main techniques for forming amyloid thin films: (i) immobilization of amyloids (Figure 2) and (ii) generating amyloid motifs from native protein thin films (Figure 3).

#### 4.1.1. Immobilization of Amyloids

The majority of amyloid fibrils have excellent mechanical strength and stick to a variety of substrates. There are a lot of methods that allow post-assembly immobilization for the production of 2D amyloid systems, such as filtration [83], stacking [84], and self-assembly at the air–water interface [85]. In a two-step process described by Knowles et al. [84], protein molecules assemble into amyloid fibrils under specific conditions, favoring intermolecular instead of intramolecular interactions (Figure 2).

The protein solution is incubated with hydrochloric acid at 65 °C for 14 days to induce self-assembly into highly stable nanofibrils. This hydrogen-bonded, dense material can be obtained from a variety of proteins and peptides. Amyloid fibrils are then cast into thin films onto a flat polymer film. After solvent evaporation, the result is a self-standing, stable protein film, which can be manipulated by tweezers. The characterization of these films by XRD has revealed a high level of order at nano- and micrometer scales. Indeed, individual strands making up the nanofibrils are at a 4.8 Å distance along the direction of the fibrils. Sheets made up of arrays of strands associate laterally to form parallel assemblies, resulting in a characteristic distance of about 12 Å in the direction perpendicular to the strand repeat. XRD also shows that the long axes of the amyloid fibrils become aligned in the film plane during casting from the hydrogel into the films. These findings further demonstrate that the fibrils’ extremely regular core structure is not affected at the atomic level by the casting and drying process. Moreover, amyloid thin films with a clearly defined hierarchy of length scales can be created using this two-step method [84].

AFM and numerical simulations of a bimodal solution containing long, semiflexible β-lactoglobulin fibrils and short, flexible β-lactoglobulin linear aggregates at an air–water interface were performed by Jordens et al. [85]. In these experiments, the conformations of fibrils and aggregates at the liquid–air interface appeared to be the result of a complex interplay between the underlying thermodynamics of the composite system, the hydrodynamics, and the non-equilibrium conditions related to the irreversible adsorption.

Qin and coworkers exploit amyloid-like protein aggregation to produce amyloid-like nanofilms within a few seconds and with strong adhesion on a hydrogel/tissue [86]. The strong adhesion with the hydrogel/tissue surface is attributed to various functional groups simultaneously exposed on the functionalized phase-transitioned human lactoferrin (*PTHLF*) nanofilm surface, especially the large number of hydrophobic groups, which effectively break through the hydration layer of the hydrogel/tissue surface.

Liu et al. have developed an amyloid-like protein aggregation technique that was inspired by naturally occurring protein amyloid fibrillization capable of assembling proteins into supramolecular 2D films with incredibly huge diameters and long-lasting interfacial adhesion stability [87]. This method opens up a novel avenue for the manufacturing of protein thin films in which the assembly of proteins is guided by spontaneous interfacial 2D aggregation of protein oligomers rather than the more conventional 1D protofibril elongation. As a result, by simply adjusting the interfacial aggregation pathways, the film shape, thickness, porosity, and function may be customized. Resulting amyloid-inspired protein thin films, as a new type of biomimetic material, provide a good platform for integration with various biomedical functions. Here, the creation of bioactive surfaces on virtually arbitrary substrates by amyloid-like protein thin films is discussed, highlighting antimicrobial, antifouling, molecular separation, and interfacial biomineralization activities that exceed those of their native protein precursors and synthetic alternatives.

#### 4.1.2. Appearance of Amyloid Motifs in Monomeric Protein Multilayers

Other methods include first protein immobilization into a thin film followed by amyloid motif generation into the film by applying specific conditions, such as, for example, thermal annealing (Figure 3). Indeed, the amyloid motif was found after monomer protein immobilization by the Langmuir–Blodgett technique on Si_3_N_4_ membranes with subsequent immediate drying in gaseous nitrogen and thermal annealing that consists of incubating the film for 10 min at 150 °C and cooling to room temperature. LB techniques used for both enzyme immobilization and crystallographic templates result in rather stable 2D protein organization [88]. Localized globular aggregates and filamentous spherulites based on nanofibrillar subunits with cross-β amyloidic patterns were observed in annealed LB multilayers [89].

**Figure 4:**
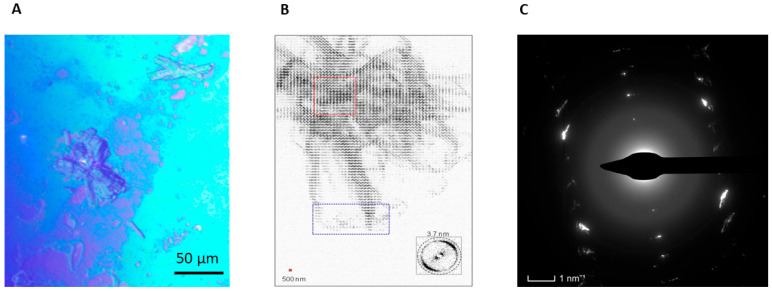
(**A**) Spherulitic structures appearing in PGA multilayered LB film after annealing at 150 °C. (**B**) Density map based on a composite of scanning nanoXRD patterns from a PGA spherulite with 500 nm (hxv) raster step-increments; The spherulitic core (dotted red rectangle) and filamentary arms extending from the core (dotted blue rectangle) are clearly distinguished. The resolution range of a pixel is shown in the inset. (**C**) Cryo-EM microED fiber texture pattern with annealed phycocyanin LB multilayers.

We propose that advanced characterization techniques such as X-ray nanodiffraction and Cryo-EM in microED mode can critically advance our understanding of amyloid formation directly in the thin film of monomeric protein. Namely, scanning x-ray nanodiffraction experiments of penicillin-G-acylase (PGA) multilayers deposited on Si_3_N_4_ membranes and annealed at 150 °C have revealed that while the annealed multilayer has remained mostly featureless, locally globular aggregates and filamentous spherulites based on nanofibrillar subunits with cross-β amyloidic motifs can be observed (Figure 4A,B) [89,90]. On the other hand, it has been demonstrated by cryo-electron microdiffraction that amorphous phycocyanin LB multilayers form, after annealing at 150 °C and cooling to room temperature, a layered nanofibrillar lattice with rotational disorder (Figure 4C) [91].

## 5. Conclusions and Future Trends

A deep comprehension of the precise structure of naturally occurring and designed amyloid fibrils and their aggregates requires studying them using cutting-edge advanced technologies. This will have a significant impact on the understanding of neurodegenerative disorders and aid in the development of innovative early diagnosis and therapeutic pathways. Assembled into thin films, amyloids can be studied by the most advanced characterization methods and then easily find application in various fields of science and technology, e.g., be employed in nanotechnological devices both for biomedical and industrial application.

However, further research should be conducted in order to clarify the selection criteria and mechanisms underlying the production of 2D and 3D materials based on amyloids. A lot of fundamental information on regulated 2D and 3D amyloid formations can be obtained by advanced technologies, which in turn are becoming more available for the scientific community.

## Data Availability

No new data were created or analyzed in this study. Data sharing is not applicable to this article.
